# Supplementary material for: SORCS1 and SORCS3 control energy balance and orexigenic peptide production
Source: EMBO Rep. 2018 Feb 12;19(4):e44810. doi: 10.15252/embr.201744810 (PMC5891432; doi:10.15252/embr.201744810)
Supplement: Supplementary file 1 — Appendix [file EMBR-19-e44810-s001.pdf]

APPENDIX

TABLE OF CONTENTS

APPENDIX SUPPLEMENTARY METHODS 2

Analysis of glucose metabolism in mice 2

Electrophysiological recording from brain slices 2

Time-lapse imaging of primary neurons 3

APPENDIX FIGURES 4

Appendix Figure S1: Generation of mice doubly deficient for *Sorcs1* and *Sorcs3* (S1/3 KO) 4

Appendix Figure S2: Expression of *Sorcs3* in the paraventricular nucleus of the mouse hypothalamus 5

Appendix Figure S3: Activity of AgRP neurons is not affected by SORCS1/3 ablation 6

SUPPLEMENTARY REFERENCES 7

APPENDIX SUPPLEMENTARY METHODS

Analysis of glucose metabolism in mice

For glucose tolerance test (GTT), overnight fasted mice were injected intraperitoneally (i. p.) with D-glucose (2 g/kg body weight), and blood was collected before and following the injection from the facial vein of non-anesthetized animals. Glucose was measured from whole blood using glucometer (Contour, Bayer). Insulin was measured using commercially available ELISA (Crystal Chem Inc. #90080). For insulin tolerance test (ITT), 5 h fasted mice were i. p. injected with human recombinant insulin (0.5 U/kg body weight; Eli Lilly) and glucose was monitored before and after the injection in tail-tip blood. For pyruvate tolerance test (PTT), overnight fasted mice were i. p. injected with sodium pyruvate (1 g/kg body weight), and tail-tip blood glucose was measured before and after the injection.

Electrophysiological recording from brain slices

Coronal slices containing the arcuate nucleus of the hypothalamus were prepared from 7 – 8 weeks old (*Sorcs1/3*$^{-/-}$; *Npy*-GFP$^{Tg/-}$) (Npy/S1/3 KO) and littermate controls (*Sorcs1/3*$^{+/+}$; *Npy*-GFP$^{Tg/-}$) (Npy/WT). Slice preparations were always performed between 10:00 – 11:00 am to be consistent with the physiological state of the animals. In brief, animals were anesthetized and decapitated. Brains were quickly removed into ice-cold sucrose containing artificial cerebral spinal fluid (S-ACSF, containing 87 mM NaCl, 26 mM NaHCO3, 10 mM glucose, 2.5 mM KCl, 1.25 mM NaH_2PO_4, 3 mM $MgCl_2$, 0.5 mM $CaCl_2$, and 50 mM sucrose, pH 7.4). Tissue blocks were mounted on a vibratome (Leica VT 1200, Leica Microsystems), cut at 300 µm thickness, and stored in an interface-type chamber. The interface chamber was perfused with storage ACSF (containing 119 mM NaCl, 26 mM $NaHCO_3$, 10 mM glucose, 2.5 mM KCl, 1 mM NaH_2PO_4, 2.5 mM $CaCl_2$, and 1.3 mM $MgCl_2$). Slices were incubated for 60 min before recordings started. All ACSF solutions were equilibrated with carbogen (95% O2 and 5% CO2).

Prior to recording, slices were transferred to a submerged recording chamber (Luigs and Neumann, Ratingen, Germany) and perfused with carbogenated ACSF (same as storage ACSF) at 32 – 34°C with a perfusion rate of 2.5–3.0 ml/min. Recording electrodes of 3–5 MΩ resistance were pulled from borosilicate glass capillaries (Harvard Apparatus, Kent, UK; 1.5 mm OD) using a micropipette electrode puller (DMG Universal Puller). AgRP positive neurons were identified by the presence of GFP autofluorescence. Biocytin (0.2%) was included in the patch pipette to assess the morphology and correct location of the recorded neurons following the experiments. The intracellular solution contained 130 mM K-gluconate, 7 mM KCl, 10 mM HEPES, 4 mM Mg-ATP, 0.3 mM Na-GTP, 10 mM Na-phosphocreatine, and 0.2% (w/v) biocytin, adjusted to pH 7.25 with KOH, approximately 300 mOsm. Cell-attached and whole-cell recordings of AgRP neurons were performed with an Axopatch 700B Amplifier (Axon Instruments, Union City, CA, USA). Data were acquired using a BNC-2090 adapter chassis, digitized (PCI 6035E A/D Board, National Instruments, Austin, Texas) at 5–10 kHz, and recorded in IGOR Pro (WaveMetrics Inc., OR, USA). GFP+ AgRP neurons were initially recorded in the cell-attached mode (voltage clamped at -60mV) to quantify the firing rate of action currents and then whole-cell configuration was achieved to characterize the firing pattern (in current clamp) and to determine the resting membrane potential.

Time-lapse imaging of primary neurons

Primary cortical neurons were plated on PDL-coated glass bottom dishes (MatTek). On DIV6, the neurons were transfected with an expression construct encoding rat TrkB fused to eGFP using Lipofectamine^{TM} 2000 reagent. Imaging was performed 24 h post-transfection on an inverted spinning disk confocal microscope (Nikon CSU-W) equipped with an electron multiplying charge-coupled device camera (ANDOR) and a temperature- and CO_2-controlled incubator. For imaging, conditioned medium in the neuronal culture was replaced by FluoroBrite^{TM} DMEM (Gibco), supplemented with recombinant BDNF (R&D Systems) at 100 ng/ml. Images were acquired every 700 ms over a period of 2 min using 40 x oil immersion objective. Kymographs were generated from time-lapse movies using KymoAnalyzer ImageJ software tool [1]. Trajectories of individual moving vesicles were manually assigned, and transport parameters were automatically calculated by KymoAnalyzer.

APPENDIX FIGURES

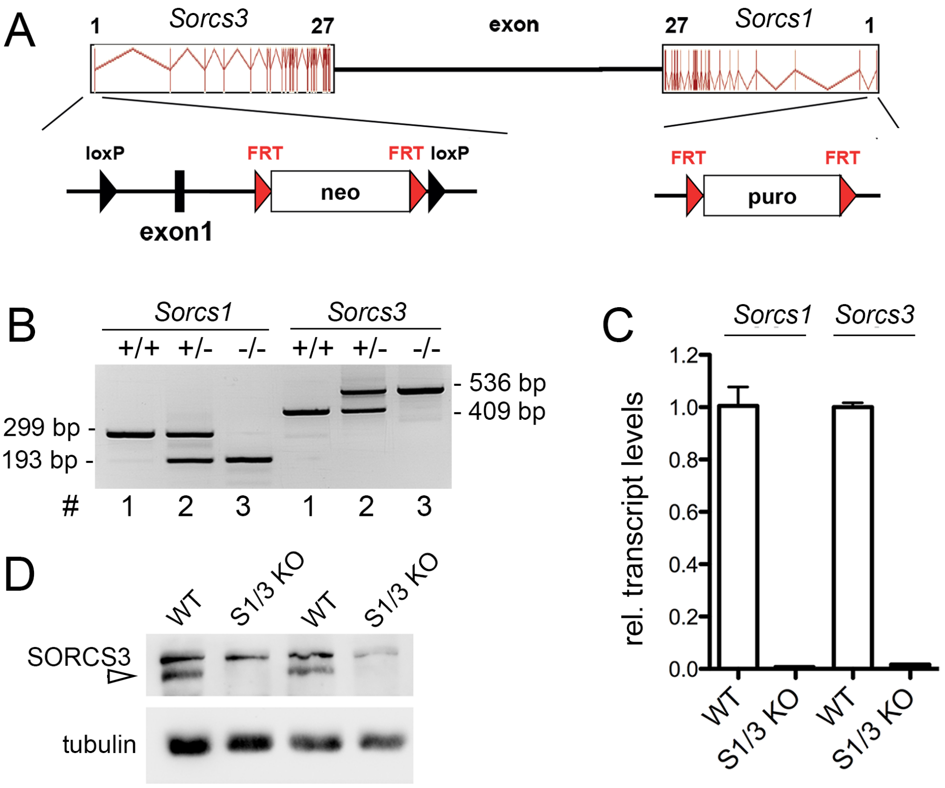

Appendix Figure S1: Generation of mice doubly deficient for *Sorcs1* and *Sorcs3* (S1/3 KO)

(A) Scheme illustrating the strategy for generation of S1/3KO mice (see Methods section for details). The localization of exons 1 through 27 in *Sorcs1* and *Sorcs3* is indicated. For targeted disruption, exons 1 in *Sorcs1* and *Sorcs3* were replaced by expression cassettes conferring resistance to puromycin (puro) or neomycin (neo), respectively.

(B) Representative genotyping PCR on genomic DNA isolated from mice either WT (#1, +/+), heterozygous (#2, +/-), or homozygous deficient (#3, -/-) for *Sorcs1* and *Sorcs3*. The sizes of the PCR products in base pairs (bp) are indicated.

(C) Quantitative RT-PCR on hippocampal RNA using primers that amplify exons 23 to 24 in *Sorcs1* or exons 5 to 6 in *Sorcs3.* Expression levels in WT animals were set to 1. Expression of *Sorcs1* and *Sorcs3* transcripts is lost in S1/3 KO. Data are shown as mean ± SD (n=3 mice/ group).

(D) Western blot analysis on membrane protein fractions of cerebral cortex lysates documents lack of SORCS3 expression in S1/3 KO mice. The immunoreactive band representing SORCS3 (130 kDa) is indicated by an arrowhead. Detection of tubulin in the protein preparations served as loading control. n=4 mice/ group.

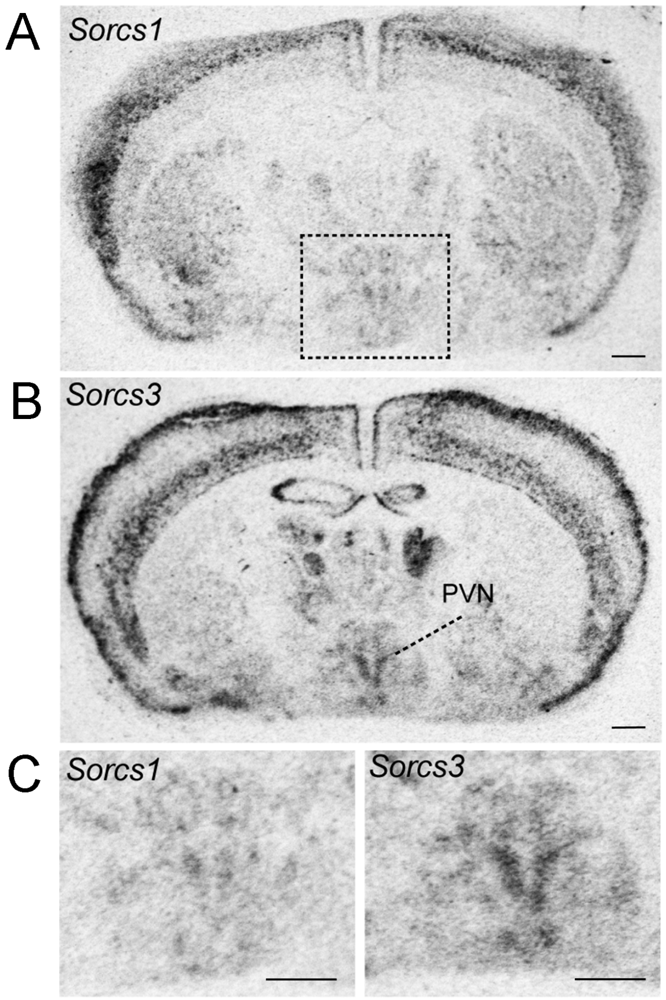

Appendix Figure S2: Expression of *Sorcs3* in the paraventricular nucleus of the mouse hypothalamus

In situ hybridization for *Sorcs1* (A) and *Sorcs3* (B) on coronal sections of the mouse brain indicating expression of *Sorcs3*, but not *Sorcs1*, in the paraventricular nucleus of the hypothalamus (PVN). Panels in C represent higher magnification of the PVN area marked in the overview micrographs in panels A and B. Scale bar: 500 µM. n=3 mice.

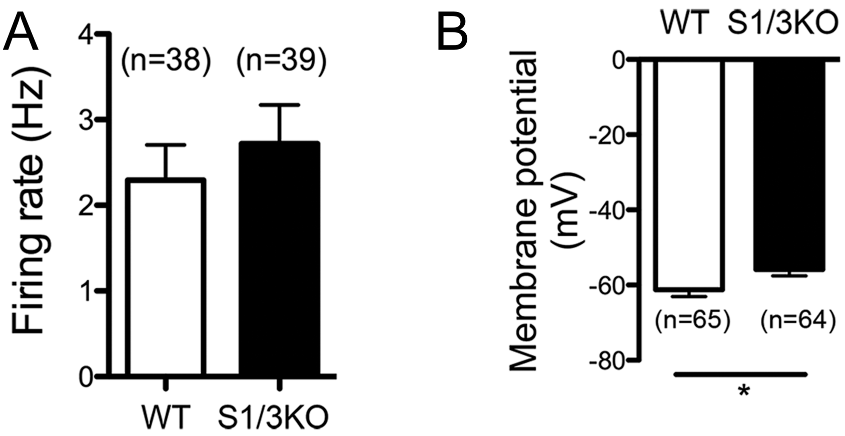

Appendix Figure S3: Activity of AgRP neurons is not affected by SORCS1/3 ablation

Firing rate (A) and membrane potential (B) of NPY/AgRP neurons were determined in brain slices from *Npy*-GFP/WT and *Npy*-GFP/S1/3 KO mice at 8 weeks of age. The number of recorded neurons is indicated in brackets. n=7 mice/ group.

SUPPLEMENTARY REFERENCES

1. Neumann S, Chassefeyre R, Campbell GE, Encalada SE (2017) KymoAnalyzer: a software tool for the quantitative analysis of intracellular transport in neurons. ***Traffic*** **18**: 71-88
