# Supplementary material for: SORCS1 and SORCS3 control energy balance and orexigenic peptide production
Source: EMBO Rep. 2018 Feb 12;19(4):e44810. doi: 10.15252/embr.201744810 (PMC5891432; doi:10.15252/embr.201744810)
Supplement: Supplementary file 2 — Expanded View Figures PDF [file EMBR-19-e44810-s002.pdf]

Expanded View Figures

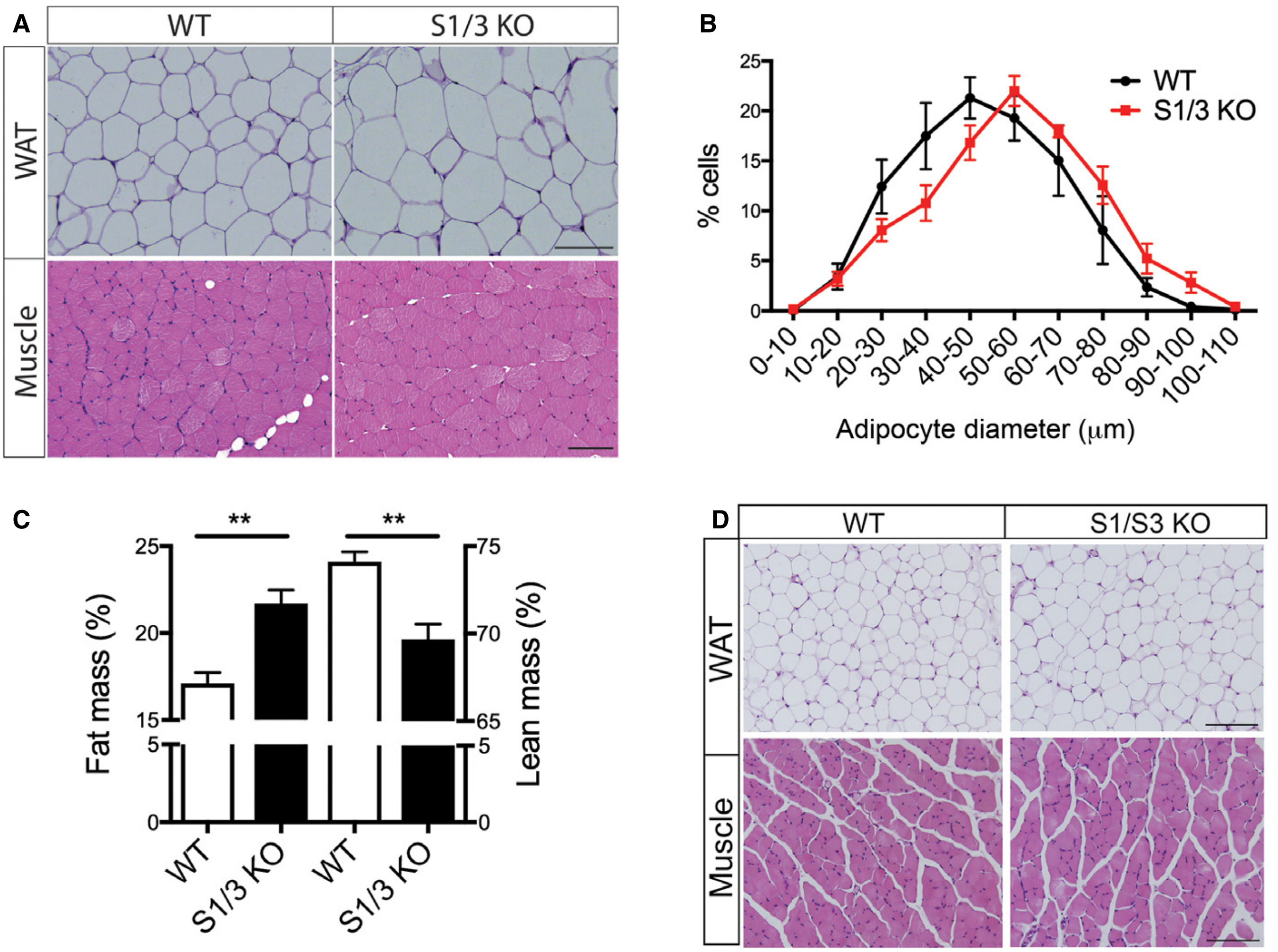

Figure EV1. Morphology of white adipose tissue and muscle in S1/3 KO mice of different ages.

A Representative hematoxylin and eosin (H&E)-stained sections of perigonadal white adipose tissue (WAT) and quadriceps muscle from 14- to 15-week-old WT and S1/3 KO mice. Scale bars: 100 μm.

B Adipocyte size distribution shows a decrease in the number of small cells and an increase in the number of large cells (% of total cell numbers) in perigonadal adipose tissue of S1/3 KO mice as compared to controls. For each mouse, 332–551 adipocytes across the tissue depot were analyzed on H&E-stained sections (n = 4–5 mice/group). Two-way ANOVA; P = 0.0207 for interaction between genotype and adipocyte size.

C Body composition as determined by NMR imaging in 6-week-old mice of the indicated genotypes. S1/3 KO mice show an increase in fat mass and a concomitant decrease in lean (fat-free) mass as compared to WT controls (n = 5–6 animals/group).

D Representative H&E-stained sections of perigonadal white adipose tissue (WAT) and quadriceps muscle from 6-week-old WT and S1/3 KO mice. Scale bars: 100 μm. n = 3–5 mice/genotype.

Data information: Data in (B and C) are shown as mean ± SEM and were analyzed using two-way ANOVA with Bonferroni post-test (B) or two-tailed unpaired t-test (C). $^{**}P < 0.01$.

© 2018 The Authors

Figure EV2. Impaired glucose tolerance in adult S1/3 KO mice on a normal chow.

A Blood glucose and insulin levels after overnight fasting in 12-week-old WT and S1/3 KO mice (n = 12–17 animals/group).

B Glucose tolerance test (GTT) in mice at 12 weeks of age. S1/3 KO mice show increased blood glucose levels after an i.p. bolus of glucose (2 g/kg body weight) as compared to WT controls (n = 15–17 mice/group).

C Area under the curve (AUC) for GTT.

D Plasma insulin levels in mice of the indicated genotype during GTT (n = 9–10 mice/group).

E Insulin tolerance test (ITT) in mice at 13 weeks of age. Blood glucose levels after an i.p. injection of insulin (0.75 U/kg body weight) were not significantly different between the genotypes (n = 8–9 mice/group).

F Pyruvate tolerance test (PTT) in mice of the indicated genotypes at 18 weeks of age. Blood glucose levels after an i.p. injection of sodium pyruvate (1 g/kg body weight) are shown (n = 8–9 mice/group).

G Area under curve (AUC) for PTT.

Data information: Data are shown as mean ± SEM and were analyzed using a two-tailed unpaired t-test (A, C, G) or two-way ANOVA with Bonferroni post-test (B, D–F). **$P < 0.01$, ***$P < 0.001$.

© 2018 The Authors

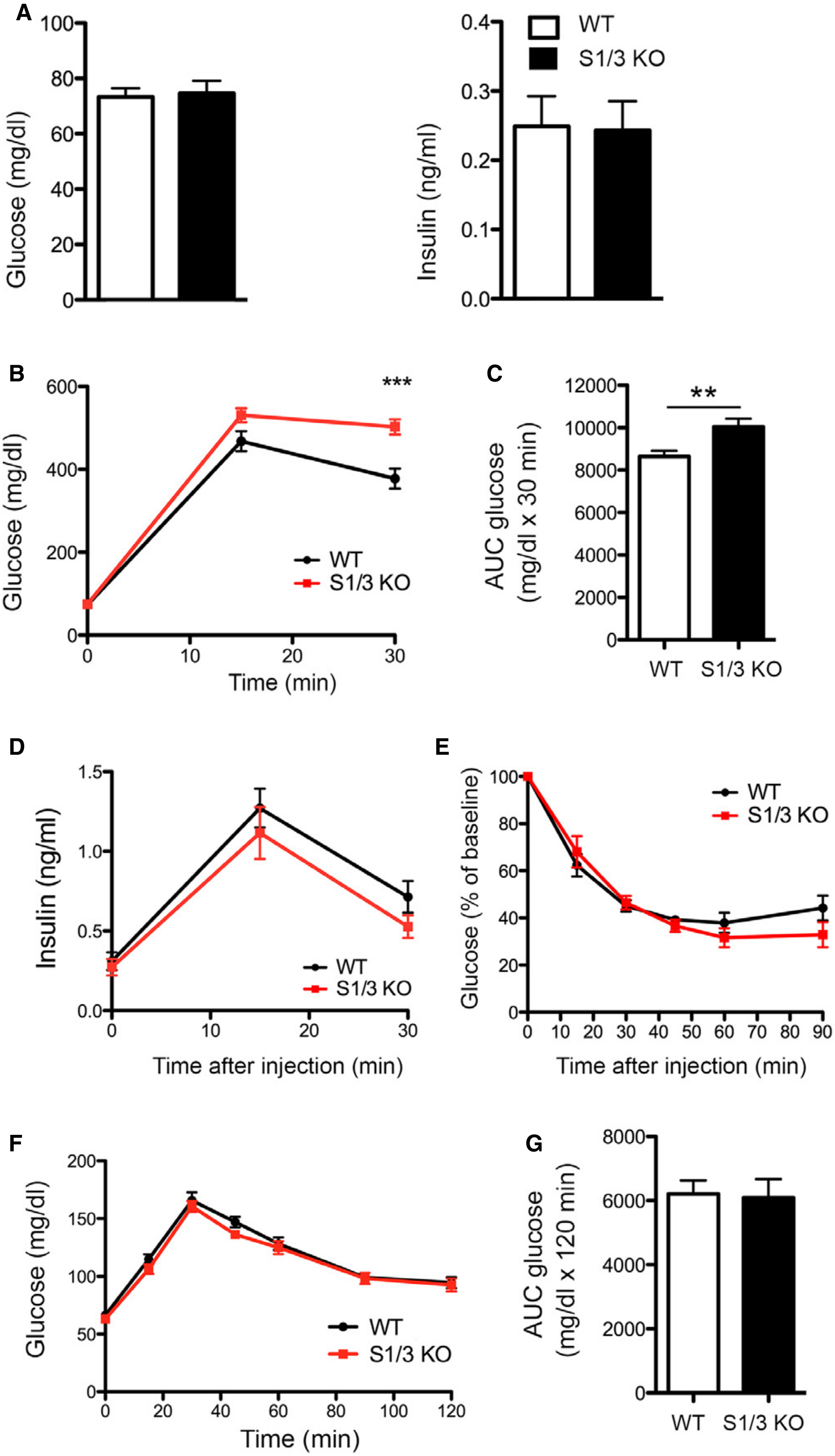
A
Glucose (mg/dl)
100
80
60
40
20
0
Insulin (ng/ml)
0.4
0.3
0.2
0.1
0.0
WT
S1/3 KO
B
Glucose (mg/dl)
600
400
200
0
***
WT
S1/3 KO
0
10
20
30
Time (min)
C
AUC glucose
(mg/dl x 30 min)
12000
10000
8000
6000
4000
2000
0
**
WT
S1/3 KO
D
Insulin (ng/ml)
1.5
1.0
0.5
0.0
WT
S1/3 KO
0
10
20
30
Time after injection (min)
E
Glucose (% of baseline)
100
80
60
40
20
0
WT
S1/3 KO
0
10
20
30
40
50
60
70
80
90
Time after injection (min)
F
Glucose (mg/dl)
200
150
100
50
0
WT
S1/3 KO
0
20
40
60
80
100
120
Time (min)
G
AUC glucose
(mg/dl x 120 min)
8000
6000
4000
2000
0
WT
S1/3 KO

Figure EV2.

Figure EV3. Glucose homeostasis in aged S1/3 KO mice.

A, B Blood glucose (A) and insulin (B) levels after overnight fasting in 9-month-old WT and S1/3 KO mice (n = 6–9 animals/group).
C Plasma insulin levels before and after an i.p. bolus of glucose (2 g/kg body weight) in 9-month-old WT and S1/3 KO mice (n = 6–9 mice/group).
D Insulin tolerance test at 9 months of age. Blood glucose levels were measured before and after an i.p. injection of insulin (0.75 U/kg body weight) (n = 7–9 mice/group).
E Glucose tolerance test (GTT) at 8 months of age. Blood glucose levels were measured before and after an i.p. bolus of glucose (2 g/kg body weight) (n = 6–8 mice/group).
F Area under curve (AUC) for GTT.

Data information: Data are shown as mean ± SEM and were analyzed using a two-tailed unpaired t-test (A, B, F) or two-way ANOVA with Bonferroni post-test (C–E). *$P < 0.05$, **$P < 0.01$.

© 2018 The Authors

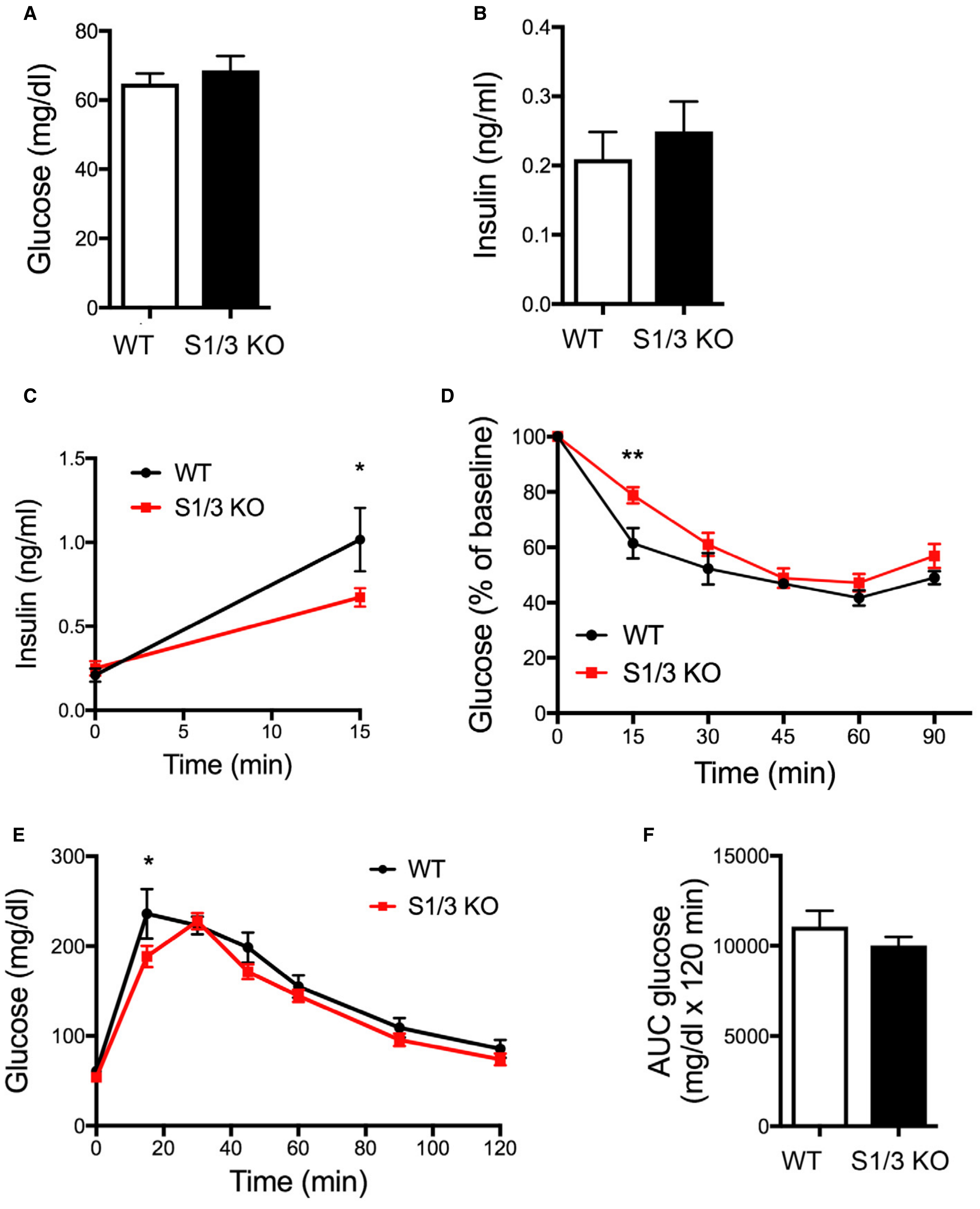
A
Glucose (mg/dl)
80
60
40
20
0
WT
S1/3 KO
B
Insulin (ng/ml)
0.4
0.3
0.2
0.1
0.0
WT
S1/3 KO
C
Insulin (ng/ml)
1.5
1.0
0.5
0.0
WT
S1/3 KO
*
0
5
10
15
Time (min)
D
Glucose (% of baseline)
100
80
60
40
20
0
**
WT
S1/3 KO
0
15
30
45
60
90
Time (min)
E
Glucose (mg/dl)
300
200
100
0
*
WT
S1/3 KO
0
20
40
60
80
100
120
Time (min)
F
AUC glucose (mg/dl x 120 min)
15000
10000
5000
0
WT
S1/3 KO

Figure EV3.

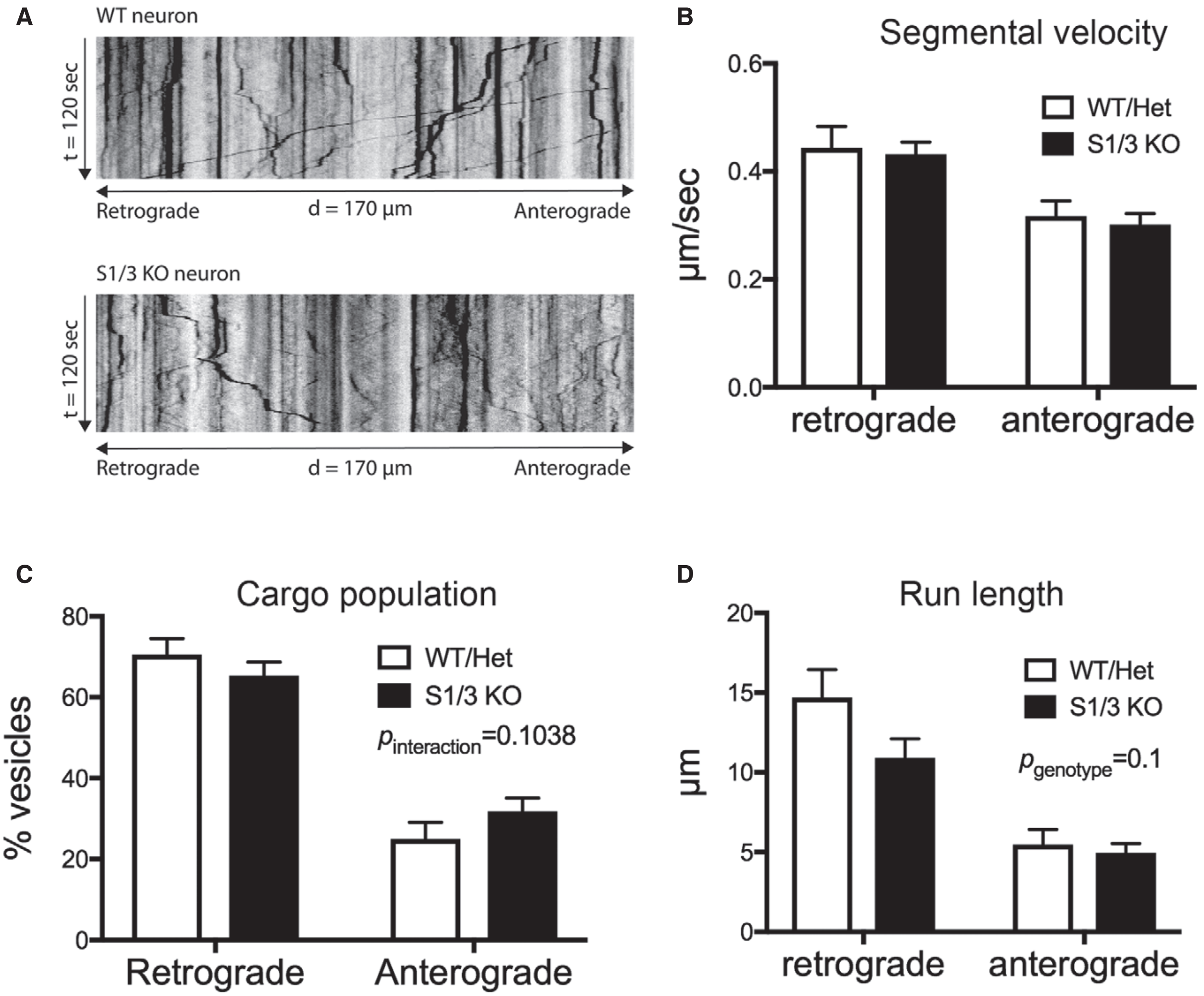

Figure EV4. Time-lapse imaging of GFP-TrkB trafficking in primary cortical neurons from WT and S1/3 KO mice.

A Representative kymographs (time–space plots) generated from time-lapse movies visualizing trafficking of transiently overexpressed GFP-TrkB in DIV7 primary cortical neurons from WT and S1/3 KO mice.

B Segmental velocity of GFP-TrkB vesicles uninterruptedly moving in retrograde or anterograde direction in axons from WT and S1/3 KO primary cortical neurons. (n = 15–20 neurons/genotype from two neuronal culture preparations each).

C Population (% of total) of GFP-TrkB vesicles moving in retrograde and anterograde directions in axons from WT and S1/3 KO primary cortical neurons (n = 28–29 neurons/genotype from three neuronal culture preparations each).

D Total distance covered by GFP-TrkB vesicles uninterruptedly moving in retrograde or anterograde directions in axons from WT and S1/3 KO primary cortical neurons. (n = 15–20 neurons/genotype from two neuronal culture preparations each).

Data information: Data in (B–D) are shown as mean ± SEM and analyzed using two-way ANOVA with Bonferroni post-test.

 © 2018 The Authors
