# Supplementary material for: SORCS1 and SORCS3 control energy balance and orexigenic peptide production
Source: EMBO Rep. 2018 Feb 12;19(4):e44810. doi: 10.15252/embr.201744810 (PMC5891432; doi:10.15252/embr.201744810)
Supplement: Supplementary file 3 — Table EV1 [file EMBR-19-e44810-s003.docx]

**Table EV1: Selected list of proteins with altered cell surface exposure in SORCS1/3 deficient neurons**

| **Protein** | | **Gene** | **Relative levels**  **S1/3 KO/ WT** | **P value** | **Function** | **Localization** |
| --- | --- | --- | --- | --- | --- | --- |
| *Neurotransmitter release* |  | | | | | |
| Vesicle-associated membrane proteins 2, 3 | | *Vamp2, 3* | 0.29 | 0.015 | synaptic transmission | secretory vesicle, cell membrane |
| Syntaxin-1A | | *Stx1a* | 0.73 | 0.048 | synaptic transmission | synaptic vesicle, cell membrane |
| Synaptotagmin-2 | | *Syt2* | 0 | 0.015 | synaptic transmission | synaptic vesicle, cell membrane |
| Synaptotagmin-11 | | *Syt11* | >10 | 0.031 | synaptic transmission | transmembrane |
| Synapsin-1 | | *Syn1* | 0.67 | <0.001 | synaptic transmission | synaptic vesicles |
| *Neuronal receptors* |  | | | | | |
| Glutamate receptor ionotropic, delta-1 | | *Grid1* | 0.25 | 0.032 | glutamate receptor | synaptic membrane |
| γ-aminobutyric acid type B receptor subunit 1 | | *Gabbr1* | 0.27 | 0.018 | GABA receptor | synaptic membrane |
| Protein NipSnap homolog 1 | | *Nipsnap1* | 0 | 0.045 | neuropeptide receptor | synaptic membrane |
| Tomoregulin-1 | | *Tmeff1* | 0 | 0.001 | BMP signaling | transmembrane |
| Amyloid precursor protein | | *App* | 1.63 | 0.009 | receptor | transmembrane |
| BDNF/NT-3 growth factor receptor | | *Ntrk2* | 1.57 | 0.021 | receptor | transmembrane |
| Neogenin | | *Neo1* | 1.35 | 0.012 | axon guidance | transmembrane |
| Transferrin receptor protein 1 | | *Tfrc* | 0.36 | <0.001 | transferrin receptor | transmembrane |
| Plexin-D1 | | *Plxnd1* | 0 | 0.008 | semaphorin signaling | transmembrane |
| Plexin-C1 | | *Plxnc1* | 0.13 | 0.037 | semaphorin signaling | transmembrane |
| *Transporters* |  | | | | | |
| Neutral amino acid transporter A | | *Slc1a4* | 0.24 | 0.004 | amino acid transport | transmembrane |
| Na/Cl-dependent GABA transporter 1 | | *Slc6a1* | 0.72 | 0.018 | GABA transporter | transmembrane |
| *Other synaptic proteins* |  | | | | | |
| Neurexin-1 | | *Nrxn1* | 1.26 | 0.031 | synaptic adhesion | transmembrane |
| Contactin-associated protein-like 2 | | *Cntnap2* | 1.25 | 0.017 | synaptic adhesion | transmembrane |
| Nectin-3 | | *Pvrl3* | 1.60 | 0.011 | synaptic adhesion | transmembrane |
| Protocadherin-8 | | *Pcdh8* | 0 | 0.001 | synaptic transmission | transmembrane |
| Drebrin | | *Dbn1* | 0 | 0.032 | synapse assembly | postsynaptic membrane |
| Disks large homolog 3 | | *Dlg3* | 0.10 | 0.002 | NMDA-r signaling | postsynaptic membrane |
| Cell adhesion molecule 2 | | *Cadm2* | 1.26 | 0.023 | synaptic adhesion | transmembrane |
| *Secreted proteins* |  | | | | | |
| Pleiotrophin | | *Ptn* | 5.78 | <0.001 | neurite growth | secreted |
| Mesenc. astrocyte-derived neurotrophic factor | | *Manf* | 2.08 | 0.002 | neurotrophic factor | secreted |
| Brevican core protein | | *Bcan* | >10 | 0.017 | axon growth | extracellular |
| Glypican-1 | | *Gpc1* | 1.45 | 0.029 | axon growth | secreted |
| *Adaptors* |  | | | | | |
| PI-binding clathrin assembly protein | | *Picalm* | 0.26 | 0.012 | endocytosis | associates with membrane proteins |
| Clathrin coat assembly protein AP180 | | *Snap91* | 0.59 | 0.004 | endocytosis | associates with clathrin coated pits |
